# Disposable e-cigarettes and their nicotine delivery, usage pattern, and subjective effects in occasionally smoking adults

**DOI:** 10.1038/s41598-025-97491-5

**Published:** 2025-05-09

**Authors:** Christin Falarowski, Elke Pieper, Andrea Rabenstein, Nadja Mallock-Ohnesorg, Nestor Burgmann, Klaas Franzen, Marcus Gertzen, Gabriele Koller, Dennis Nowak, Anna Rahofer, Benedikt Rieder, Gabriel Roselli de Oliveira Pinto Kise, Thomas Schulz, Elena Strohmeyer, Peter Laux, Andreas Luch, Tobias Rüther

**Affiliations:** 1 Department of Psychiatry and Psychotherapy, LMU University Hospital, LMU Munich, Munich, Germany; 2 Department of Chemical and Product Safety, German Federal Institute for Risk Assessment (BfR), Berlin, Germany; 3 Institute of Legal Medicine, Goethe-University Frankfurt, Kennedyallee 104, 60596 Frankfurt/Main, Germany; 4 Medical Clinic III, University Hospital Schleswig-Holstein, Campus Lübeck, 23562 Lübeck, Germany; 5 Airway Research Center North, Member of the German Center for Lung Research (DZL), 22927 Großhansdorf, Germany; 6 Department of Psychiatry, Psychotherapy and Psychosomatics, Faculty of Medicine, University of Augsburg, Geschwister-Schönert-Str. 1, 86159 Augsburg, Germany; 7 Institute of Occupational, Social and Environmental Medicine, CPC (Comprehensive Pneumology Center) Munich, LMU University Hospital, LMU Munich, DZL (Deutsches Zentrum für Lungenforschung), Munich, Germany

**Keywords:** Psychiatric disorders, Disease prevention

## Abstract

New disposable e-cigarettes are popular, especially among young people. This study aimed to assess their addictive potential by examining puffing behaviors, subjective effects, and conducting laboratory investigations in an experimental setting. Single-center, four-arm, crossover study. Laboratory setting in Munich (LMU-University Hospital), Germany. Eighteen occasionally cigarette-smoking adults aged 19–28. Two disposable e-cigarettes of the same brand (strawberry-kiwi/ tobacco flavored) were tested with a cigarette and a conventional pod e-cigarette as comparators. Usage was ad libitum for 5 min (or the duration of smoking one cigarette) while puffing topography was recorded. Distributed over 30 min blood samples for nicotine plasma concentrations were drawn and hemodynamic parameters and subjective effects assessed. Nicotine delivery of disposable e-cigarettes (C_max_ strawberry-kiwi: 7.1 ng/ml, tobacco: 6.9 ng/ml) was similar to the cigarette (8.1 ng/ml) and higher than for the pod e-cigarette (3.1 ng/ml). The nicotine plasma increase in the first minute was the fastest for disposable e-cigarettes. T_max_ for the disposable e-cigarettes (strawberry-kiwi: 5 min, tobacco: 6 min) was reached faster than for the cigarette (8 min). Compared to the pod e-cigarette (6 min), t_max_ was reached faster with the disposable e-cigarette tobacco. Total nicotine uptake was the lowest for the pod e-cigarette. Puffing behavior differed between e-cigarettes and the cigarette and slightly between both disposable e-cigarettes. Satisfaction and motivation to consume the product again were higher for disposables with a slight preference for strawberry-kiwi flavor. New disposable e-cigarettes hold great addictive potential for young people unestablished in smoking. Besides conducting further research, it is crucial to observe usage trends and strengthen regulations.

Trial registration number: DRKS00030978. Date of registration: 21.02.2023.

**Supplementary Information:**

The online version contains supplementary material available at 10.1038/s41598-025-97491-5.

## Introduction

The prevalence of electronic cigarette (e-cigarette) use is high among young people and increasing worldwide^1,2^.

There has been a noticeable shift towards new disposable e-cigarettes, as seen in Germany^3^ or in the USA^4^. Moreover, disposables were reported as the most used device type among 13- to 40-year-old ever-users of e-cigarettes in the United States^5^. A British survey reports an 18-fold increase in the percentage of adult disposable users from 2021 to 2022 with the most rapid growth in the youngest age group (18 years old)^6^. Adolescents in the UK perceive disposable e-cigarettes as a ‘fashionable’ lifestyle ‘accessory’^7^.

A recent study raised safety concerns, as the investigated disposable was found to contain high levels of nicotine, flavor chemicals, and synthetic coolants whose inhalation risks are still unknown^8^.

E-cigarettes are offered with a great variety of over 7000 estimated different flavors^9^. The initiation of e-cigarette use and intention to start cigarette smoking among adolescents is more likely through flavored e-cigarettes^10,11^. Fruit-flavored e-cigarettes are also perceived as less harmful^12^.

This perception is dangerous since Electronic Nicotine Delivery System (ENDS) liquids and aerosols can, for example, contain toxicants or carcinogens besides the highly addictive nicotine^13^. The nicotine concentration in e-liquids is legally limited to 20 mg/ml in the European Union while e-liquids usually contain significantly more nicotine in the USA^14,15^.

Metanalysis has shown that e-cigarettes can be a useful tool for current cigarette smokers to quit or at least reduce their individual health risks^16^. Cigarette smoke contains significantly more harmful substances than e-cigarette aerosol and is accountable for 13.6% of deaths worldwide^17,72^. However, in this paper, the focus is on the addictiveness and smoking initiation potential of e-cigarettes. E-cigarette use should not be confused with being harmless.

Tobacco is a highly addictive substance with a dependence potential ranking directly after heroin and cocaine^18^. Psychological and physical factors lead to tobacco dependence and its maintenance^19^. One major aspect is the speed of nicotine increasing in blood that differs between nicotine products^20^: Faster delivery leads to greater rewarding subjective effects and reinforcing behavior^21,22^. A rapid increase is associated with higher addictiveness^20,22^. Among other factors, the efficient nicotine delivery profile makes smoking the most addictive, reinforcing form of nicotine administration^20,23,24^. The highest measured nicotine plasma concentration (C_max_) in the plasma after smoking one cigarette typically ranges from 10 to 30 ng/ml^25^.

The nicotine delivery profile of e-cigarettes depends on the specific device design (such as power), liquid composition (nicotine concentration), operation conditions, and user experience^26^. It is possible for people who use e-cigarettes to produce a bolus cigarette-like delivery profile with a sharp plasma nicotine increase^25,27^. However, this applied to a study with a puffing regime, while a study with 90 min ad libitum use of e-cigarettes reported varying nicotine curve shapes with an overall slower increase over time. The authors suggest there are implications for a lower abuse liability compared with cigarettes^25,28^.

Studies reporting nicotine plasma concentrations during e-cigarette use show deviating results depending on the device. First-generation devices (“cig-a-likes”) were mostly found to have inefficient or negligible nicotine delivery. However, one study reported an average C_max_ of 10.3 ng/ml, which is in the range of a cigarette^25,29^. Advanced generation e-cigarettes showed significantly higher nicotine levels compared to first-generation e-cigarettes^30,31^. Nevertheless, they did not reach the efficiency of cigarettes^30,31^.

Published research addressed aspects such as the prevalence of use^6,32^, perception and engagement among youth^7^, liquid and device characteristics^8,33^, patterns of sales^34^, or the harm reduction potential of disposable e-cigarettes^35^, but their pharmacokinetic (PK) or addictive potential has been hardly studied up to this point^33,35^. Thus, the presented four-arm crossover study focused on nicotine delivery, puffing behavior, and subjective effects of disposable e-cigarettes in comparison with a cigarette and a pod e-cigarette. Additionally, hemodynamic and negative subjective effects were measured. To investigate the effect of flavor, the disposable e-cigarette was included in two study arms, one using a fruit flavor and the other using a tobacco flavor. Due to ethical considerations, people who do not smoke were not included in the study. Instead, people who smoke occasionally were recruited as subjects, as the primary research focus was on the initiation of e-cigarette use, rather than regular smoking behavior. To our knowledge, this is the first study to examine the new disposable e-cigarettes in this kind of population.

Fearon et al.^35^ investigated nicotine PKs of a new disposable e-cigarette (BIDI^®^ Stick) in six various flavors in comparison with a cigarette and a pod e-cigarette in eighteen adults who smoke. Consumption was mixed with a puffing regime followed by ad libitum use. The results showed that the disposable e-cigarette was as effective in nicotine delivery as the cigarette and more effective than the pod e-cigarette. Subjective effects for relief, satisfaction, and aversion were similar for all products. While they focused on the possible role of disposable e-cigarettes in smoking cessation as an effective alternative^35^, we aimed to assess the addictiveness of e-cigarettes among people nearly unestablished in smoking to gain information concerning the initiation of e-cigarette use and acute effects.

Another recent study compared liquid characteristics and e-cigarette dependence between pod e-cigarettes and disposable e-cigarettes^33^. The authors conducted an online study among people who (formerly) used e-cigarettes aged 18 to 65. The results support the hypothesis of an association between nicotine flux and nicotine dependence among people who use pod e-cigarettes but did not show the same for disposable e-cigarettes. Also, they did not find an association between device power or nicotine concentration and e-cigarette dependence. Nicotine flux and nicotine concentration were generally higher for disposables than for pod e-cigarettes. The nicotine flux of many disposables reported in the study also exceeded the nicotine flux of a cigarette. This raises concerns as high nicotine flux may increase the abuse liability of a product^26^. Device power of all disposables was in the medium range while it broadly varied for pod e-cigarettes but was mainly in the low range^33^.

As the described studies touch on topics discussed in our study but with different aims and methods, our study is a valuable contribution to research regarding new disposable e-cigarettes. The main study objective was to assess the addictive potential and risk of the investigated disposable e-cigarette for young occasional smokers to start consumption compared to the cigarette and pod e-cigarette. We expected the disposable e-cigarette to facilitate the initiation of use compared to the other products. Therefore, we assessed nicotine delivery, puffing behavior, hemodynamic and subjective effects. The detailed hypotheses stated before execution of the study are listed in the supplement.

## Materials and methods

The study was performed in accordance with the principles of the Declaration of Helsinki and approved by the Ethical Committee at the Medical Faculty of Ludwig-Maximilians-University Munich (No. 22-1069). Registration took place at the “Bundesinstitut für Arzneimittel und Medizinprodukte” (BfArM) (DRKS00030978). Written informed consent was obtained prior to participation. Travel and subject insurance were covered by HDI Global SE (No. 39 130537 03026/ 03440).

Four products were tested by each participant in randomized order determined by a programmed tool:

- Disposable e-cigarette strawberry-kiwi: Elf Bar 600 Strawberry Kiwi (20 mg/ml nicotine) by iMiracle Shenzhen Technology Co. Ltd.
- Disposable e-cigarette tobacco: Elf Bar 600 Tobacco (20 mg/ml nicotine) by iMiracle Shenzhen Technology Co. Ltd.
- Cigarette: Marlboro Red cigarette (0.8 mg nicotine) by Philip Morris.
- Pod e-cigarette: Myblu tobacco roasted blend (18 mg/ml nicotine) by Reemtsma.

Nicotine levels refer to product information. Products were purchased online and in stores in Germany from January to May 2023. The Elf Bar was chosen because of its popularity^36,37^. We decided on strawberry-kiwi flavor on the one hand to represent fruit-flavor which adolescents seem to be especially interested in trying^12^ and on the other hand tobacco flavor to represent a flavor similar to the cigarette and the pod e-cigarette for comparison.

18 adults (18 to 29 years old) were recruited via social media. To determine the sample size a power calculation was performed. It was estimated based on a partial eta square. All participants smoked at least once a month, up to 3 days per week, and were not tobacco dependent according to ICD-10, Fagerström Test for Cigarette Dependence (FTCD)^38–40^ or Penn State Electronic Cigarette Dependence Index (PSECDI)^41^. People who exceeded the inclusion criteria at any time in the past were excluded. Nicotine abstinence was required at least 12 h before an examination.

The study was performed from February to July 2023 at LMU University Hospital, Department of Psychiatry and Psychotherapy, Munich, Germany. After being screened and receiving information on the study subjects completed four sessions with one product per visit.

All study visits were performed indoors with the possibility of smoke/vapor removal by opening a window. Participants were seated during the session. Consumption was ad libitum meaning participants could use a product freely without any puffing regime. The given time frame for consumption was five min for pod e-cigarettes and disposable e-cigarettes. The use time for the cigarette ended when one cigarette was finished regardless of duration. At predefined times, blood samples were taken, hemodynamic parameters were measured, and questionnaires were completed. The testing period was 30 min. The study design is visualized in the supplement. There was no training session or presentation of study products beforehand in order to avoid distortion of acute subjective effects. A picture of the study setup can be found in the supplement.

The Modified E-Cigarette Evaluation Questionnaire (mCEQ)^42^ examines subjective effects of smoking on a 7-point Likert-type scale while the Comparison Of Effects After Consumption Questionnaire (ENK)^43–45^ served to assess the negative subjective effects of smoking/e-cigarette use on a scale from 1 to 10.

Each questionnaire was filled out pre consumption, post consumption, and 30 min after initiating consumption. Immediately after smoking or e-cigarette use, participants had to rate their motivation to directly consume the tested product again on a scale from 1 to 7.

Blood samples were collected using an indwelling venous catheter at nine given times: at baseline (0 min) and 1, 2, 4, 6, 8, 10, 12, and 30 min after starting the consumption. The catheter was placed on the non-dominant hand whenever possible while the participant used the other hand for holding the respective device.

The Smoking Puff Analyzer – Mobile (SPA-M, Sodim, ORT, France) was used to examine the smoking/e-cigarette use topography^46,47^. This portable device was interposed between the cigarette or e-cigarette and the mouth for the whole consumption time. It recorded the flow rate, pressure drop profile, and atmospheric pressure of smoking. The number of puffs, puff volume, puff duration, average flow per puff, peak flow per puff, the interval between puffs, and the smoking duration were analyzed using the software SodAfc41 – Version 4.02.0 (Sodim, ORT, France).

Each average value is calculated per participant and puff whereas the respective mean average value is calculated from all 18.

Hemodynamic parameters have been assessed at baseline (0 min) as well as 7, 15, and 30 min after starting the consumption. The utilized device is the validated Mobil-O-Graph™ (software version HMS CS 4.2, I.E.M. GmbH) for measurement and calculation of heart rate, central blood pressure, peripheral blood pressure, and parameters of arterial stiffness (total vascular resistance (TVR), pulse wave velocity (PWV), and the augmentation index adjusted for heart rate 75 bpm (AIX@75)).

As PK parameters, the C_max_, total plasma nicotine uptake (area under the curve for the testing period of 30 min = AUC_0−30 min_, calculated with the linear trapezoidal rule), and the time of C_max_ (t_max_) were calculated.

SPSS26 was used for statistical analyses. Means, standard deviations, and ranges were calculated for participant characteristics. For puffing behavior and subjective effects, a repeated measures ANOVA was employed, and the according p-value for within-subject effects was considered. Pairwise t-tests for comparisons between products were calculated subsequently.

For C_max_ and AUC, geometric means and coefficients of variation were determined, and two-sided, paired t-tests with logarithmized values were performed. For t_max_, median and range were determined followed by analysis via two-sided, paired t-tests.

The statistical analysis for the cardiovascular parameters was carried out after verification of the normal distribution via the Kolmogorov-Smirnov test via two-sided, paired t-tests within the parameters against the baseline, via an ANOVA at the individual time points between the products. In addition, a two-way repeated measures ANOVA, based on baseline measurements, was used to estimate a correlation between the product used and time.

More detailed information and additional p-values (including non-significant values) can be found in the supplement.

## Results

Participants’ mean age was 24 (± 2.1), 9 being female and 9 being male. The mean number of days on which participants had smoked within the last 30 days was 4.1 (± 2.6).

### Nicotine delivery and plasma nicotine concentrations

Higher mean levels of C_max_ were reached in the cigarette (*p* < 0.001), disposable e-cigarette strawberry-kiwi (*p* < 0.001), and disposable e-cigarette tobacco (*p* < 0.001) arms compared with the pod e-cigarette. T_max_ was reached faster with the disposable e-cigarette strawberry-kiwi (*p* = 0.047) and the disposable e-cigarette tobacco (*p* = 0.012) than with the cigarette. T_max_ was also reached faster with the disposable e-cigarette tobacco than with the pod e-cigarette (*p* = 0.027). Regarding the comparison of t_max_, the difference in mean total smoking duration between the cigarette and the e-cigarette must also be considered (see below). The AUC_0−30 min_ was higher for the cigarette than for the disposable e-cigarette strawberry-kiwi (*p* < 0.007). It was at the lowest for the pod e-cigarette: significantly lower than for disposable e-cigarettes and the cigarette (*p* < 0.003 compared to the disposable e-cigarette strawberry-kiwi; *p* < 0.001 compared to cigarette and disposable e-cigarette tobacco). There were no significant differences in C_max_ (*p* = 0.88), t_max_ (*p* = 0.95), and AUC_0−30 min_ (*p* = 0.93) between both disposable e-cigarettes. The explained parameters are listed in Table 1.

**Table 1:** Summary of relevant PK parameters for nicotine (9 measurements per visit (4/648 measurements missed), C_max_ and AUC as geometric means with a coefficient of variation (CV in %); t_max_ as median and range).

Product	Disposable e-cigarette strawberry-kiwi	Disposable e-cigarette tobacco	Cigarette	Pod e-cigarette
C_max_ (ng/mL)	7.1 (168%)	6.9 (155%)	8.1 (159%)	3.1 (141%)
t_max_ (min)	5 (1–10)	6 (2–10)	8 (4–30)	6 (1–12)
AUC_0−30 min_ (ng/mL**×**h)	111.3 (143%)	110.6 (126%)	148.1 (141%)	58.3 (148%)

**Fig. 1:**
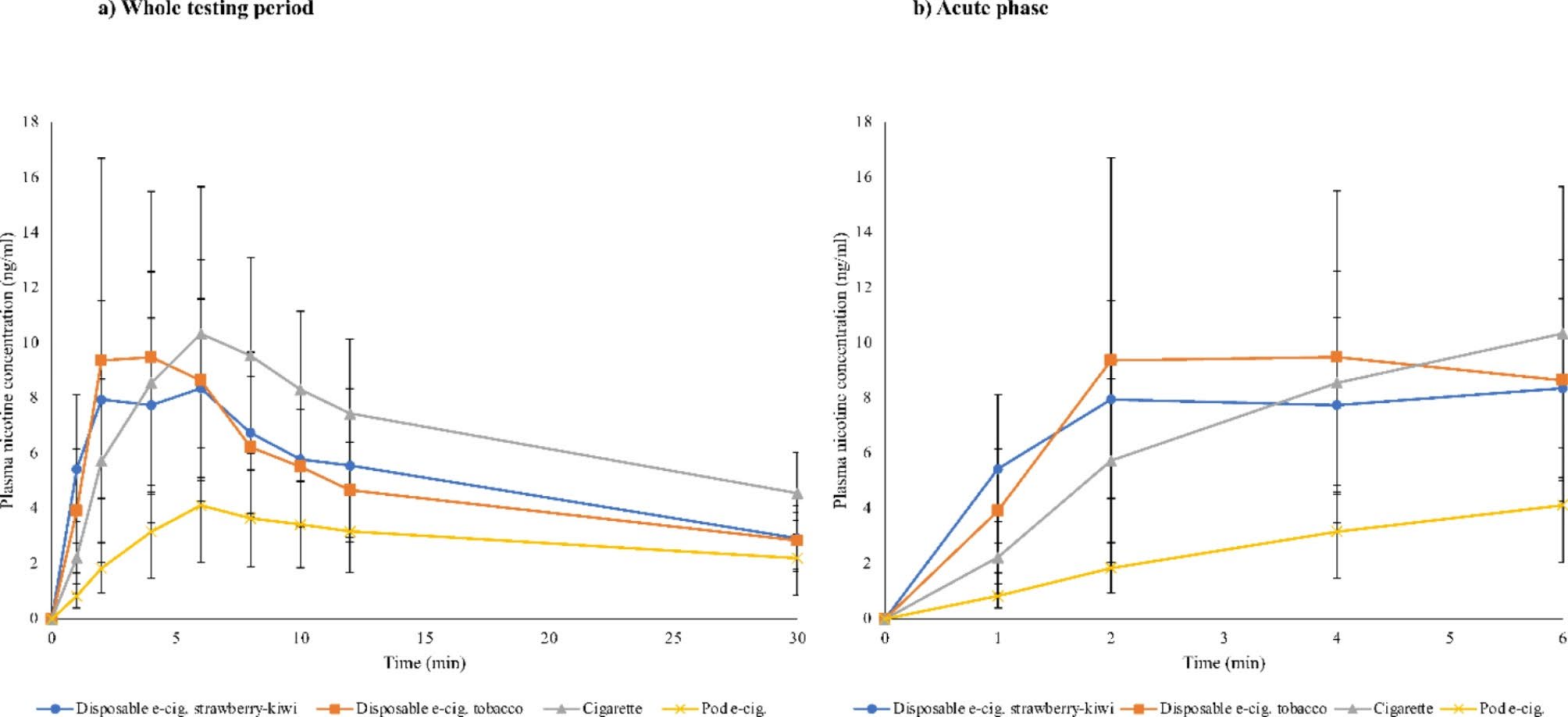
Plasma nicotine curves (arithmetic mean and 95% confidence interval (CI)).

Mean plasma nicotine concentrations as well as a magnification of the acute phase (plasma concentrations in the first six min after start of consumption) are displayed in Figs. 1a) and 1b).

1 min after the start of consumption, mean nicotine levels had increased faster for disposables than for cigarettes (*p* < 0. 03 for comparison of nicotine concentrations) and the pod e-cigarette (*p* < 0.01).

### Puffing behavior

**Fig. 2:**
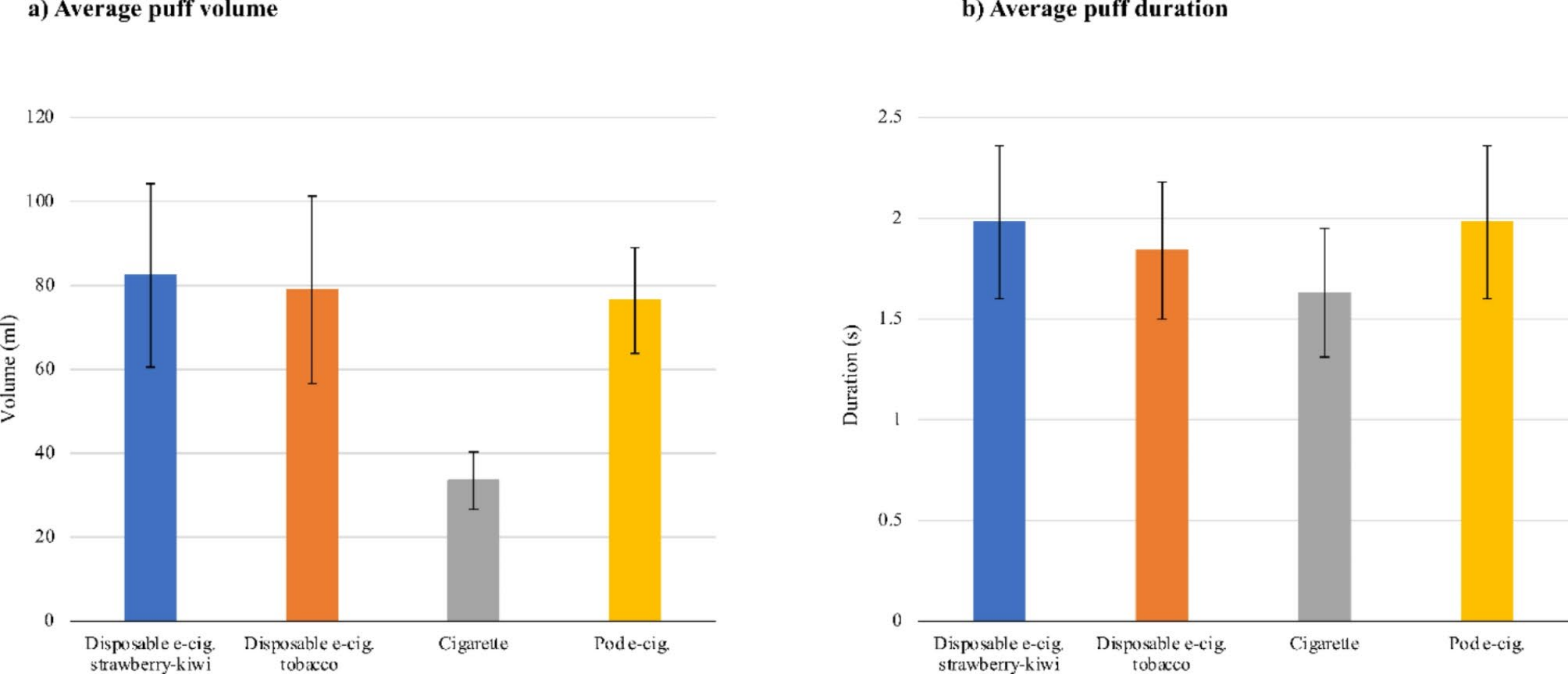
Selected values for puffing behavior (mean and 95% CI).

The mean average puff volume, as well as the mean average puff duration, were significantly higher for the disposable e-cigarette strawberry-kiwi (*p* < 0.001 for volume, *p* < 0.001 for duration), the disposable e-cigarette tobacco (*p* < 0.001 for volume, *p* = 0.024 for duration) and the pod e-cigarette (*p* < 0.001 for volume, *p* = 0.005 for duration) than for the cigarette. They are displayed in Fig. 2. Moreover, the mean puff duration was higher for the disposable e-cigarette strawberry-kiwi than for disposable e-cigarette tobacco (*p* = 0.034). The mean average puff frequency was also higher for disposable e-cigarette tobacco (*p* < 0.001), the pod e-cigarette (*p* < 0.001), and the disposable e-cigarette strawberry-kiwi (*p* < 0.001) than for the cigarette. Similarly, the mean average flow per puff and the mean peak flow per puff were higher for the disposable e-cigarette strawberry-kiwi (*p* < 0.001 for average flow, *p* < 0.001 for peak flow), disposable e-cigarette tobacco (*p* < 0.001 for average flow, *p* < 0.001 for peak flow) and the pod e-cigarette (*p* < 0.001 for average flow, *p* < 0.001 for peak flow) than for the cigarette. The mean average intervals between puffs were significantly higher for the cigarette compared with all other products (*p* = 0.01 compared with disposable e-cigarette strawberry-kiwi and pod e-cigarette, *p* < 0.001 compared with disposable e-cigarette tobacco). The mean total smoking duration was higher for the cigarette compared with the given time frame for using e-cigarettes (*p* < 0.001 for disposable e-cigarette strawberry-kiwi and pod e-cigarette, *p* < 0.001 for disposable e-cigarette tobacco). From these results, it was construed that the total number of puffs did not significantly differ between the products.

In summary, values for puffing behavior showed significant differences between e-cigarettes and cigarettes, but no significant differences between disposable e-cigarettes and pod e-cigarette. The only difference between both disposable e-cigarettes was in puff duration. The explained parameters are listed in Table 2.

**Table 2:** Summary of relevant parameters for puffing behavior in mean with 95% CI.

Product	Disposable e-cigarette strawberry-kiwi	Disposable e-cigarette tobacco	Cigarette	Pod e-cigarette
Average puff volume (ml)	82.4 (60.6-104.3)	78.9 (56.5-101.4)	33.5 (26.7–40.4)	76.4 (63.8–89.1)
Average puff duration (s)	1.98 (1.6–2.36)	1.84 (1.5–2.17)	1.63 (1.31–1.95)	1.98 (1.6–2.36)
Average flow per puff (ml/s)	42.1 (33.8–50.4)	43 (34.9–51.1)	22.8 (18.6–27.1)	41.8 (35.5–48.2)
Average peak flow per puff (ml/s)	63.1 (51.3–74.8)	62.3 (50.9–73.7)	37.5 (30.3–44.8)	64.8 (55.4–74.2)
Puff frequency (n/min)	3.49 (3.11–3.87)	3.65 (3.06–4.25)	2.76 (2.42–3.1)	3.58 (3.03–4.12)
Average interval between puffs (s)	16.12 (14.03–18.21)	16.28 (13.47–19.09)	21.34 (18.54–24.13)	16.41 (13.73–19.08)
Total duration (s)	292.96 (284.35-301.56)	293.28 (288.57–297)	345.67 (320.57-370.77)	290.55 (285.68-295.42)
Total number of puffs (n)	17.1 (15.1–19.2)	17.9 (14.9–21)	15.7 (13.9–17.4)	17.3 (14.6–20)

### Subjective effects

According to the mCEQ consumption of the disposable e-cigarette strawberry-kiwi was the most satisfying (*p* = 0.007 compared to disposable e-cigarette tobacco; *p* < 0.001 compared to cigarette; *p* < 0.001 compared to pod e-cigarette), followed by the disposable e-cigarette tobacco (*p* = 0.024 compared to cigarette; *p* = 0.01 compared to pod e-cigarette). Satisfaction scored lowest for the cigarette and the pod e-cigarette (*p* = 0.627 for comparison of cigarette and pod e-cigarette). Enjoyment of respiratory tract sensations was higher regarding the disposable e-cigarette strawberry-kiwi than for the cigarette (*p* = 0.005) and the pod e-cigarette (*p* < 0.001) with no significant difference between both disposable e-cigarettes (*p* = 0.7). Aversion to the cigarette was higher than to the pod e-cigarette (*p* = 0.026). Psychological reward and craving reduction demonstrated no significant effects between the products.

**Table 3:** Summary of mCEQ subscale results (rated directly after consumption and 30 min after the start of consumption) by mean with 95% CI.

Product	Disposable e-cigarette strawberry-kiwi	Disposable e-cigarette tobacco	Cigarette	Pod e-cigarette
Satisfaction	4.5 (4.1–4.9)	3.7 (3-4.3)	2.7 (1.9–3.5)	2.5 (1.8–3.2)
Enjoyment of respiratory tract sensations	3.5 (3–4)	3 (2.3–3.6)	2.6 (2-3.3)	2 (1.4–2.6)
Psychological reward	3.2 (2.7–3.7)	3 (2.5–3.4)	3 (2.5–3.4)	3 (2.5–3.5)
Craving reduction	5 (4.2–5.8)	4.6 (3.7–5.5)	5.2 (4.4-6)	5.2 (4.4-6)
Aversion	3.2 (2.5–3.9)	3.1 (2.6–3.6)	3.6 (2.8–4.4)	3 (2.4–3.6)

The motivation to directly consume the tested product again is illustrated in the supplement. It was significantly higher for the disposable e-cigarette strawberry-kiwi (2.9) and the disposable e-cigarette tobacco (2.7) than for the pod e-cigarette (1.5, *p* = 0.005 compared to disposable e-cigarette strawberry-kiwi and *p* = 0.006 when compared to disposable e-cigarette tobacco) and the cigarette (1.3, *p* = 0.001 compared to disposable e-cigarette strawberry-kiwi and disposable e-cigarette tobacco). It was generally low ranging from “does not apply at all” (cigarette) to “does rather not apply” (disposable e-cigarette strawberry-kiwi and e-cigarette tobacco). The explained parameters are listed in Table 3.

### Hemodynamic parameters

The peripheral as well as the central hemodynamics showed comparable results, so that in all experimental conditions significant increases for both systolic and diastolic blood pressure could be shown compared to baseline (*p* < 0.05, mostly *p* < 0.01), except for the peripheral systolic value in the disposable e-cigarette strawberry-kiwi group after 30 min with no significant difference in the mean values of the measurement time points (*p* > 0.05). For the heart rate, there was a significant difference in the mean values at any time point compared to the baseline of the time in the follow-up period with an increase in the mean values (*p* < 0.05). A comparable result was shown for the parameters of arterial vascular stiffness (TVR, PWV, and AIx@75 – *p* < 0.05 and *p* < 0.01), wherein two time points showed higher mean values of the groups without any significant difference (TVR – disposable e-cigarette strawberry-kiwi after 30 min *p* = 0.085, PWV – cigarette after 30 min *p* = 0.053). A comparison of the mean values for the individual time points between the experimental conditions did not reveal any statistically significant differences (*p* > 0.05). When looking at the results for the repeated measures ANOVA, there were no significant differences observed for the experimental condition, but there was a significant difference for all examined parameters for the time course (*p* < 0.001) and the heart rate, taking into account the experimental condition and the elapsed time (*p* < 0.05).

### Negative subjective effects

A summary of negative subjective effects and adverse events can be found in the supplement. The mean expression of negative subjective effects according to ENK was determined to be low to moderate.

## Discussion

The main finding of this study is the nicotine delivery profile of the investigated disposable e-cigarettes which is similar to the cigarette and more effective than for the pod e-cigarette. The higher scores in satisfaction and motivation to consume the product again showed the subjective preference for disposables. Puffing behavior was different for e-cigarettes and the cigarette.

Nicotine delivery is significantly associated with users’ experience. Plasma nicotine increase is higher for people who are experienced in e-cigarette use than for people who smoke but are e-cigarette-naïve^48,49^. People experienced in e-cigarette use in a real-world environment using their own device can reach effective nicotine delivery profiles comparable to cigarette smoking^25^, but this may increase their e-cigarette dependence potential^49^. An alarming conclusion is that in our study population of people who occasionally smoke with little to no e-cigarette experience or adjustment time to e-cigarette use^50^, the new disposable e-cigarettes immediately showed a highly effective nicotine delivery and uptake comparable to the cigarette. In the present study, the increase in nicotine plasma concentrations after 1 min was faster for disposable e-cigarettes than for the cigarette and the pod e-cigarette. Considering the plasma nicotine curves during the acute phase, our findings imply the highest nicotine-mediated addictive potential for the new disposable e-cigarette among the tested products.

The C_max_ for disposable e-cigarettes and cigarette did not significantly differ whereas the C_max_ for the pod e-cigarette was exceeded by far. The AUC_0−30 min_ for disposable e-cigarettes was also remarkably higher than for the pod e-cigarette. In the present study, disposable e-cigarettes seem to be more effective in regards to nicotine delivery and potentially have a higher abuse liability compared to conventional pod e-cigarettes. Regarding nicotine delivery, they are much more effective than first-generation disposables.

The AUC_0−30 min_ for the cigarette was still higher than for the disposable e-cigarette strawberry-kiwi. Nevertheless, it should be noted that the total duration was significantly higher for smoking one cigarette compared to the predetermined time frame for e-cigarette use. It cannot be ruled out that a similar amount of nicotine would have been reached with the disposable e-cigarette strawberry-kiwi within the same time taken for the smoking of one cigarette.

Puffing topography is conflicting in terms of cigarettes and e-cigarettes. Literature has reported longer puff duration^51,52^ and shorter intervals between puffs^52^ for e-cigarettes which our findings are in alignment with.

Underlining the different puffing behavior of e-cigarettes and cigarettes, our findings concerning average puff volume, puff frequency, as well as average flow and peak flow per puff were significantly higher for all e-cigarettes than for the cigarette. While the larger puff volume for e-cigarettes is in alignment with a previous study^53^, the puff velocity was reported to be lower in the respective study. This difference is likely explained by the study population of people experienced in e-cigarette use. As longer puffs lead to an increased nicotine yield in e-cigarette use but greater velocity only occurs in cigarette smoking, they may have learned to not expend the energy needed for high-velocity puffs^54^.

Noteworthy here is that there was no significant difference in puffing topography between disposable e-cigarettes and the pod e-cigarette. Consequently, it can be assumed that device design and liquid composition led to the evident difference in nicotine delivery^26^. Further arguments for the greater threat to public health of the new disposable e-cigarettes are provided by Talih et al., who investigated the design of different disposable e-cigarettes in comparison to a pod e-cigarette (not including the brands used in this study). They found lower construction quality in disposable e-cigarettes without a microcontroller circuit for temperature and power control, as well as higher nicotine concentrations as labeled. Moreover, they reported a greater emission of carcinogenic carbonyls, e.g. formaldehyde and acetaldehyde, as compared to the pod e-cigarette^55^.

The slightly higher puff duration for the disposable e-cigarette strawberry-kiwi compared with the disposable e-cigarette tobacco is indicative of adapted puffing behavior caused by flavors which has previously been reported for people experienced in e-cigarette use^56^. The authors suggest differences in subjective liking, nicotine-, and sensory effects being related to lower liquid pH as an explanation^56^.

The presented findings on subjective effects support the assumption of disposable e-cigarettes being more appealing for people who occasionally smoke compared to a pod e-cigarette and a cigarette with slight indications of preference for the strawberry-kiwi flavored disposable. Their attractiveness despite possible health risks^57–59^ can be dangerous for youth. The slight preference for the flavored e-cigarette seen in this study is supported by previous findings^60–63^. However, this is problematic since ingredients used in e-cigarettes to create flavors (including tobacco flavor) can pose a health risk^64^.

In our study, aversion was significantly higher for the cigarette vs. the pod e-cigarette which can partly be explained by the much higher nicotine uptake. The low scores for psychological reward and alternatively the scores for craving reduction without significant differences between the products as well as the general tendency to low/neutral scores in subjective measurements might have been caused by study population selection. People who smoke occasionally may not feel nicotine-induced reward in the same way as people with nicotine-dependency would and their craving for a cigarette was resolved by each product – possibly because there was no noteworthy craving to start with.

In terms of side effects, hemodynamic effects were mainly expected to be triggered by nicotine, although the effects of different flavors were still unclear. Overall, the results, even if only acute effects after a single use were investigated in this work, point in a comparable direction, as preliminary studies have already shown^65–67^. One study for example, comparing a pod-based e-cigarette with a cigarette showed a similar negative effect on cerebral and peripheral vascular function for both conditions^68^.

There is not only an increase in heart rate and blood pressure values but also alterations in the area of arterial stiffness. Consequential damage after long-term consumption based among others on the pathologically altered surrogate parameters of arterial stiffness can lead to major cardiovascular events^69^. Mechanistically, in addition to the activation of the autonomic nervous system via nicotine, the direct effect of vapor, as shown and postulated in the work of Lee et al.^70^, with a corresponding inflammatory reaction and increased reactive oxygen species, remains to be discussed. Further measurements, also directly in the area of endothelial dysfunction, which would be expected based on inflammation, would be expedient.

To conclude, our results provide an indication that the tested disposable e-cigarettes carry a high addictive potential for young people nearly unestablished in smoking. This is reflected in their effective nicotine delivery profile comparable to cigarettes. It was significantly more effective compared to a pod e-cigarette, although puffing behavior did not differ significantly between e-cigarettes, likely due to device design^26,55^. Higher scores for satisfaction and motivation to immediately re-consume the product in the disposable e-cigarette arms underline these objective results with subjective findings showing a slight preference for the strawberry-kiwi flavored disposable.

### Limitations

Further investigations with larger sample sizes are needed to confirm our findings. Other limitations are the high number of exclusions due to technical reasons and practical deviations during testing (like delays in measurements, positioning of the blood pressure cuff, or distraction of participants from consumption during blood sampling) also influencing participant comfort. Incidentally, there was likely a misunderstanding concerning the completion of the ENK: some participants reported a nicotine flash at baseline without having consumed nicotine beforehand. The study setting was not reflective of a real-life scenario as many people who occasionally smoke tend to smoke in social situations or simultaneously with alcohol consumption^71^. In this study, they consumed nicotine alone during the daytime and without the cofactors associated with their usual smoking behavior. Furthermore, conclusions about people who do not smoke should be drawn with great caution, as they are not directly comparable to people who occasionally smoke. Since we investigated on acute effects, we did not monitor participants after the last study visit. Consequently, we cannot provide information on how their usage behavior changed after the study which would indeed provide a valuable insight. Similarly, we cannot report on how the usage profile of the first tested disposable may differ from the second one or how puffing behavior evolved from the first to the last puff during one visit. Noteworthy also as a limitation is the difference in mean total duration of use which was imposed by the study design. Further, the duration of five minutes that we imposed for e-cigarette use does not correspond to the usual usage pattern of e-cigarettes. This time frame remained constant because we focused on the acute effects of consumption. A study on pod e-cigarettes, cigarettes and heated tobacco products with ad libitum consumption for 90 min among experienced users was previously performed by our study group^44^.

## Conclusion

Despite e-cigarettes’ potential usefulness in terms of smoking cessation, the conceivable role of new disposable e-cigarettes as regards acquiring dependence is alarming. With this in mind, it is necessary to observe disposable e-cigarette use trends and strengthen regulations including marketing-, flavor- or even product bans. Moreover, further research is needed to expand the knowledge about disposable e-cigarettes, usage patterns, and health-related risks.

## Electronic supplementary material

Below is the link to the electronic supplementary material.

Supplementary Material 1

## Data Availability

Data is available on request from the authors (contact information: Christin.Falarowski@med.uni-muenchen.de).

## References

[1] Society for Adolescent Health and Medicine. Protecting youth from the risks of electronic cigarettes. *J. Adolesc. Health*. **66**, 127–131. 10.1016/j.jadohealth.2019.10.007 (2020). 31780385 10.1016/j.jadohealth.2019.10.007

[2] Yoong, S. L. et al. Prevalence of electronic nicotine delivery systems (ENDS) use among youth globally: a systematic review and meta-analysis of country level data. Aust N Z J Public Health 42, 303-308. https://doi.org:10.1111/1753-6405.12777 (2018). 10.1111/1753-6405.12777 29528527

[3] Klosterhalfen, S., Viechtbauer, W. & Kotz, D. Disposable e-cigarettes: prevalence of use in Germany from 2016 to 2023 and associated user characteristics. *Addiction* **120**, 557–567. 10.1111/add.16675 (2025). 39488907 10.1111/add.16675 PMC11813731

[4] Park-Lee, E. et al. Notes from the field: E-Cigarette and nicotine pouch use among middle and high school Students - United States, 2024. *MMWR Morb Mortal. Wkly. Rep.* **73**, 774–778. 10.15585/mmwr.mm7335a3 (2024). 39236021 10.15585/mmwr.mm7335a3 PMC11376506

[5] McCauley, D. M., Gaiha, S. M., Lempert, L. K., Halpern-Felsher, B. & Adolescents Young adults, and adults continue to use E-Cigarette devices and flavors two years after FDA discretionary enforcement. *Int. J. Environ. Res. Public. Health*. **19**10.3390/ijerph19148747 (2022). 10.3390/ijerph19148747 PMC9322506 35886599

[6] Tattan-Birch, H., Jackson, S. E., Kock, L., Dockrell, M. & Brown, J. Rapid growth in disposable e-cigarette vaping among young adults in great Britain from 2021 to 2022: a repeat cross-sectional survey. *Addiction* **118**, 382–386. 10.1111/add.16044 (2023). 36065820 10.1111/add.16044 PMC10086805

[7] Smith, M. J., MacKintosh, A. M., Ford, A. & Hilton, S. Youth’s engagement and perceptions of disposable e-cigarettes: a UK focus group study. *BMJ Open.* **13**, e068466. 10.1136/bmjopen-2022-068466 (2023). 36948552 10.1136/bmjopen-2022-068466 PMC10040067

[8] Omaiye, E. E., Luo, W., McWhirter, K. J., Pankow, J. F. & Talbot, P. Disposable puff bar electronic cigarettes: chemical composition and toxicity of E-liquids and a synthetic coolant. *Chem. Res. Toxicol.* **35**, 1344–1358. 10.1021/acs.chemrestox.1c00423 (2022). 35849830 10.1021/acs.chemrestox.1c00423 PMC9382667

[9] Zhu, S. H. et al. Four hundred and sixty brands of e-cigarettes and counting: implications for product regulation. *Tobacco Control* 23, iii3-iii9 (2014). 10.1136/tobaccocontrol-2014-051670 10.1136/tobaccocontrol-2014-051670 PMC4078673 24935895

[10] Zare, S., Nemati, M. & Zheng, Y. A systematic review of consumer preference for e-cigarette attributes: flavor, nicotine strength, and type. *PLoS One*. **13**, e0194145. 10.1371/journal.pone.0194145 (2018). 29543907 10.1371/journal.pone.0194145 PMC5854347

[11] Dai, H. & Hao, J. Flavored electronic cigarette use and smoking among youth. *Pediatrics* **138**10.1542/peds.2016-2513 (2016). 10.1542/peds.2016-2513 27940718

[12] Pepper, J. K., Ribisl, K. M. & Brewer, N. T. Adolescents’ interest in trying flavoured e-cigarettes. *Tob. Control*. **25** (ii62-ii66). 10.1136/tobaccocontrol-2016-053174 (2016). 10.1136/tobaccocontrol-2016-053174 PMC5125087 27633762

[13] Walley, S. C., Jenssen, B. P. & Section on Tobacco Control. *Electron. Nicotine Delivery Syst. Pediatr.* **136**, 1018–1026 10.1542/peds.2015-3222 (2015). 10.1542/peds.2015-3222 26504128

[14] Snell, L. M. et al. Emerging electronic cigarette policies in European member States, Canada, and the united States. *Health Policy*. **125**, 425–435. 10.1016/j.healthpol.2021.02.003 (2021). 33663799 10.1016/j.healthpol.2021.02.003 PMC8025686

[15] European Commission. *Questions & Answers: New rules for tobacco products* (2014). https://europa.eu/rapid/press-release_MEMO-14-134_en.htm

[16] Hartmann-Boyce, J. et al. Electronic cigarettes for smoking cessation. *Cochrane Database Syst. Reviews*. 10.1002/14651858.CD010216.pub7 (2022). 10.1002/14651858.CD010216.pub4 PMC8094228 33052602

[17] Benowitz, N. L., Helen, S., Liakoni, E. & G. & Clinical Pharmacology of electronic nicotine delivery systems (ENDS): implications for benefits and risks in the promotion of the combusted tobacco endgame. *J. Clin. Pharmacol.* **61** (Suppl 2), S18–s36. 10.1002/jcph.1915 (2021). 34396553 10.1002/jcph.1915 PMC9239851

[18] Nutt, D., King, L. A., Saulsbury, W. & Blakemore, C. Development of a rational scale to assess the harm of drugs of potential misuse. *Lancet* **369**, 1047–1053. 10.1016/s0140-6736(07)60464-4 (2007). 17382831 10.1016/S0140-6736(07)60464-4

[19] Sohn, M., Hartley, C., Froelicher, E. S. & Benowitz, N. L. Tobacco use and dependence. *Semin Oncol. Nurs.* **19**, 250–260. 10.1053/j.soncn.2003.08.002 (2003). 14702859 10.1053/j.soncn.2003.08.002

[20] Henningfield, J. E. & Keenan, R. M. Nicotine delivery kinetics and abuse liability. *J. Consult Clin. Psychol.* **61**, 743–750. https://doi.org/10.1037/0022-006x.61.5.743 (1993). 10.1037//0022-006x.61.5.743 8245272

[21] de Wit, H., Bodker, B. & Ambre, J. Rate of increase of plasma drug level influences subjective response in humans. *Psychopharmacol. (Berl)*. **107**, 352–358. 10.1007/bf02245161 (1992). 10.1007/BF02245161 1615136

[22] Benowitz, N. L. Nicotine addiction. *Prim. Care*. **26**, 611–631. 10.1016/s0095-4543(05)70120-2 (1999). 10436290 10.1016/s0095-4543(05)70120-2

[23] Hukkanen, J., Jacob, P., Benowitz, N. L. & 3rd & Metabolism and disposition kinetics of nicotine. *Pharmacol. Rev.* **57**, 79–115. 10.1124/pr.57.1.3 (2005). 15734728 10.1124/pr.57.1.3

[24] Benowitz, N. L., Porchet, H. & Jacob, P. in *Nicotine Psychopharmacology: Molecular, Cellular, and Behavioural Aspects* (eds S. Wonnacott, M. A. H. Russell, & I. P. Stolerman) 0Oxford University Press (1990).

[25] Voos, N., Goniewicz, M. L. & Eissenberg, T. What is the nicotine delivery profile of electronic cigarettes? *Expert Opin. Drug Deliv*. **16**, 1193–1203. 10.1080/17425247.2019.1665647 (2019). 31495244 10.1080/17425247.2019.1665647 PMC6814574

[26] Shihadeh, A. & Eissenberg, T. Electronic cigarette effectiveness and abuse liability: predicting and regulating nicotine flux. *Nicotine Tob. Res.* **17**, 158–162. 10.1093/ntr/ntu175 (2015). 25180079 10.1093/ntr/ntu175 PMC4837999

[27] St Helen, G., Havel, C., Dempsey, D. A., Jacob, P., Benowitz, N. L. & 3rd & Nicotine delivery, retention and pharmacokinetics from various electronic cigarettes. *Addiction* **111**, 535–544. 10.1111/add.13183 (2016). 26430813 10.1111/add.13183 PMC4749433

[28] St Helen, G. et al. Nicotine delivery and vaping behavior during ad libitum E-cigarette access. *Tob. Regul. Sci.* **2**, 363–376. 10.18001/trs.2.4.8 (2016). 28393086 10.18001/TRS.2.4.8 PMC5381821

[29] Vansickel, A. R. & Eissenberg, T. Electronic cigarettes: effective nicotine delivery after acute administration. *Nicotine Tob. Res.* **15**, 267–270. 10.1093/ntr/ntr316 (2013). 22311962 10.1093/ntr/ntr316 PMC3524053

[30] Hajek, P., Przulj, D., Phillips, A., Anderson, R. & McRobbie, H. Nicotine delivery to users from cigarettes and from different types of e-cigarettes. *Psychopharmacol. (Berl)*. **234**, 773–779. 10.1007/s00213-016-4512-6 (2017). 10.1007/s00213-016-4512-6 PMC5306435 28070620

[31] Voos, N. et al. Randomized within-subject trial to evaluate smokers’ initial perceptions, subjective effects and nicotine delivery across six vaporized nicotine products. *Addiction* **114**, 1236–1248. 10.1111/add.14602 (2019). 30851137 10.1111/add.14602 PMC6646880

[32] Wang, T. W. et al. Disposable E-Cigarette use among U.S. Youth - An emerging public health challenge. *N Engl. J. Med.* **384**, 1573–1576. 10.1056/NEJMc2033943 (2021). 33725431 10.1056/NEJMc2033943

[33] Do, E. K. et al. E-cigarette device and liquid characteristics and E-cigarette dependence: A pilot study of pod-based and disposable E-cigarette users. *Addict. Behav.* **124**, 107117. 10.1016/j.addbeh.2021.107117 (2022). 34555560 10.1016/j.addbeh.2021.107117 PMC8511126

[34] Diaz, M. C., Silver, N. A., Bertrand, A. & Schillo, B. A. Bigger, stronger and cheaper: growth in e-cigarette market driven by disposable devices with more e-liquid, higher nicotine concentration and declining prices. *Tob. Control*. 10.1136/tc-2023-058033 (2023). 37536928 10.1136/tc-2023-058033 PMC11877113

[35] Fearon, I. M., Gilligan, K., Seltzer, R. G. N. & McKinney, W. A randomised, crossover, clinical study to assess nicotine pharmacokinetics and subjective effects of the BIDI(^®^) stick ENDS compared with combustible cigarettes and a comparator ENDS in adult smokers. *Harm Reduct. J.* **19**, 57. 10.1186/s12954-022-00638-0 (2022). 35655314 10.1186/s12954-022-00638-0 PMC9160848

[36] Birdsey, J. et al. Tobacco product use among U.S. Middle and high school Students - National youth tobacco survey, 2023. *MMWR Morb Mortal. Wkly. Rep.* **72**, 1173–1182. 10.15585/mmwr.mm7244a1 (2023). 37917558 10.15585/mmwr.mm7244a1 PMC10629751

[37] East, K. et al. Use of ‘elf bar’ among youth and young adults who currently vape in England: cross-sectional associations with demographics, dependence indicators and reasons for use. *Addiction* **120**, 414–422. 10.1111/add.16463 (2025). 38515247 10.1111/add.16463 PMC11415542

[38] Fagerstrom, K. O. & Schneider, N. G. Measuring nicotine dependence: a review of the Fagerstrom tolerance questionnaire. *J. Behav. Med.* **12**, 159–182. 10.1007/bf00846549 (1989). 2668531 10.1007/BF00846549

[39] Heatherton, T. F., Kozlowski, L. T., Frecker, R. C. & Fagerström, K. O. The Fagerström test for nicotine dependence: a revision of the Fagerström tolerance questionnaire. *Br. J. Addict.* **86**, 1119–1127. 10.1111/j.1360-0443.1991.tb01879.x (1991). 1932883 10.1111/j.1360-0443.1991.tb01879.x

[40] Fagerström, K. Determinants of tobacco use and renaming the FTND to the Fagerstrom test for cigarette dependence. *Nicotine Tob. Res.* **14**, 75–78. 10.1093/ntr/ntr137 (2012). 22025545 10.1093/ntr/ntr137

[41] Foulds, J. et al. Development of a questionnaire for assessing dependence on electronic cigarettes among a large sample of ex-smoking E-cigarette users. *Nicotine Tob. Res.* **17**, 186–192. 10.1093/ntr/ntu204 (2015). 25332459 10.1093/ntr/ntu204 PMC4838001

[42] Cappelleri, J. C. et al. Confirmatory factor analyses and reliability of the modified cigarette evaluation questionnaire. *Addict. Behav.* **32**, 912–923. 10.1016/j.addbeh.2006.06.028 (2007). 16875787 10.1016/j.addbeh.2006.06.028

[43] Vukas, J. et al. Two different heated tobacco products vs. Cigarettes: comparison of nicotine delivery and subjective effects in experienced users. *Toxics* **11**10.3390/toxics11060525 (2023). 10.3390/toxics11060525 PMC10301154 37368625

[44] Rabenstein, A. et al. Usage pattern and nicotine delivery during ad libitum consumption of pod E-Cigarettes and heated tobacco products. *Toxics* **11**10.3390/toxics11050434 (2023). 10.3390/toxics11050434 PMC10221897 37235249

[45] Mallock, N. et al. Nicotine delivery and relief of craving after consumption of European JUUL e-cigarettes prior and after pod modification. *Sci. Rep.* **11**, 12078. 10.1038/s41598-021-91593-6 (2021). 34103661 10.1038/s41598-021-91593-6 PMC8187405

[46] Mikheev, V. B. et al. The application of commercially available mobile cigarette topography devices for E-cigarette vaping behavior measurements. *Nicotine Tob. Res.* **22**, 681–688. 10.1093/ntr/nty190 (2018). 10.1093/ntr/nty190 PMC7171281 30215774

[47] Vansickel, A. R. et al. Characterization of puff topography of a prototype electronic cigarette in adult exclusive cigarette smokers and adult exclusive electronic cigarette users. *Regul. Toxicol. Pharmacol.* **98**, 250–256. 10.1016/j.yrtph.2018.07.019 (2018). 30053435 10.1016/j.yrtph.2018.07.019

[48] Hiler, M. et al. Electronic cigarette user plasma nicotine concentration, puff topography, heart rate, and subjective effects: influence of liquid nicotine concentration and user experience. *Exp. Clin. Psychopharmacol.* **25**, 380–392. 10.1037/pha0000140 (2017). 29048187 10.1037/pha0000140 PMC5657238

[49] Farsalinos, K. E. et al. Nicotine absorption from electronic cigarette use: comparison between experienced consumers (vapers) and Naïve users (smokers). *Sci. Rep.* **5**, 11269. 10.1038/srep11269 (2015). 26082330 10.1038/srep11269 PMC4469966

[50] Lee, Y. H., Gawron, M. & Goniewicz, M. L. Changes in puffing behavior among smokers who switched from tobacco to electronic cigarettes. *Addict. Behav.* **48**, 1–4. 10.1016/j.addbeh.2015.04.003 (2015). 25930009 10.1016/j.addbeh.2015.04.003 PMC4457608

[51] Farsalinos, K. E., Romagna, G., Tsiapras, D., Kyrzopoulos, S. & Voudris, V. Evaluation of electronic cigarette use (vaping) topography and Estimation of liquid consumption: implications for research protocol standards definition and for public health authorities’ regulation. *Int. J. Environ. Res. Public. Health*. **10**, 2500–2514. 10.3390/ijerph10062500 (2013). 23778060 10.3390/ijerph10062500 PMC3717749

[52] Strasser, A. A. et al. Nicotine replacement, topography, and smoking phenotypes of E-cigarettes. *Tob. Regul. Sci.* **2**, 352–362. 10.18001/trs.2.4.7 (2016). 27942543 10.18001/TRS.2.4.7 PMC5142626

[53] Spindle, T. R., Breland, A. B., Karaoghlanian, N. V., Shihadeh, A. L. & Eissenberg, T. Preliminary results of an examination of electronic cigarette user puff topography: the effect of a mouthpiece-based topography measurement device on plasma nicotine and subjective effects. *Nicotine Tob. Res.* **17**, 142–149. 10.1093/ntr/ntu186 (2015). 25239957 10.1093/ntr/ntu186 PMC4838000

[54] Talih, S. et al. Effects of user puff topography, device voltage, and liquid nicotine concentration on electronic cigarette nicotine yield: measurements and model predictions. *Nicotine Tob. Res.* **17**, 150–157. 10.1093/ntr/ntu174 (2015). 25187061 10.1093/ntr/ntu174 PMC4837998

[55] Talih, S. et al. Electrical features, liquid composition and toxicant emissions from ‘pod-mod’-like disposable electronic cigarettes. *Tob. Control*. **31**, 667–670. 10.1136/tobaccocontrol-2020-056362 (2022). 33980722 10.1136/tobaccocontrol-2020-056362 PMC8586044

[56] St Helen, G., Shahid, M., Chu, S. & Benowitz, N. L. Impact of e-liquid flavors on e-cigarette vaping behavior. *Drug Alcohol Depend.* **189**, 42–48. 10.1016/j.drugalcdep.2018.04.032 (2018). 29879680 10.1016/j.drugalcdep.2018.04.032 PMC6211798

[57] Lin, H. C., Buu, A. & Su, W. C. Disposable E-Cigarettes and associated health risks: an experimental study. *Int. J. Environ. Res. Public. Health*. **19**10.3390/ijerph191710633 (2022). 10.3390/ijerph191710633 PMC9518067 36078349

[58] McGrath-Morrow, S. A. et al. The effects of nicotine on development. *Pediatrics* **145**10.1542/peds.2019-1346 (2020). 10.1542/peds.2019-1346 PMC7049940 32047098

[59] Rosenthal, H., Chow, N., Mehta, S., Pham, D. & Milanaik, R. Puff bars: a dangerous trend in adolescent disposable e-cigarette use. *Curr. Opin. Pediatr.* **34**, 288–294. 10.1097/mop.0000000000001123 (2022). 35152232 10.1097/MOP.0000000000001123

[60] Audrain-McGovern, J., Strasser, A. A. & Wileyto, E. P. The impact of flavoring on the rewarding and reinforcing value of e-cigarettes with nicotine among young adult smokers. *Drug Alcohol Depend.* **166**, 263–267. 10.1016/j.drugalcdep.2016.06.030 (2016). 27426010 10.1016/j.drugalcdep.2016.06.030 PMC4995771

[61] Soule, E. K., Lopez, A. A., Guy, M. C. & Cobb, C. O. Reasons for using flavored liquids among electronic cigarette users: A concept mapping study. *Drug Alcohol Depend.* **166**, 168–176. 10.1016/j.drugalcdep.2016.07.007 (2016). 27460860 10.1016/j.drugalcdep.2016.07.007 PMC4983519

[62] Soneji, S. S., Knutzen, K. E. & Villanti, A. C. Use of flavored E-Cigarettes among adolescents, young adults, and older adults: findings from the population assessment for tobacco and health study. *Public. Health Rep.* **134**, 282–292. 10.1177/0033354919830967 (2019). 30857471 10.1177/0033354919830967 PMC6505324

[63] Goldenson, N. I. et al. Effects of sweet flavorings and nicotine on the appeal and sensory properties of e-cigarettes among young adult vapers: application of a novel methodology. *Drug Alcohol Depend.* **168**, 176–180. 10.1016/j.drugalcdep.2016.09.014 (2016). 27676583 10.1016/j.drugalcdep.2016.09.014 PMC5086287

[64] Ween, M. P. et al. E-cigarettes and health risks: more to the flavor than just the name. *Am. J. Physiol. Lung Cell. Mol. Physiol.* **320**, L600–l614. 10.1152/ajplung.00370.2020 (2021). 33295836 10.1152/ajplung.00370.2020 PMC8424560

[65] Gernun, S. et al. Cardiovascular functions and arterial stiffness after JUUL use. *Tob. Induc. Dis.* **20**, 34. 10.18332/tid/144317 (2022). 35431721 10.18332/tid/144317 PMC8973023

[66] Franzen, K. F. et al. E-cigarettes and cigarettes worsen peripheral and central hemodynamics as well as arterial stiffness: A randomized, double-blinded pilot study. *Vasc Med.* **23**, 419–425. 10.1177/1358863x18779694 (2018). 29985113 10.1177/1358863X18779694

[67] Antoniewicz, L., Brynedal, A., Hedman, L., Lundbäck, M. & Bosson, J. A. Acute effects of electronic cigarette inhalation on the vasculature and the conducting airways. *Cardiovasc. Toxicol.* **19**, 441–450. 10.1007/s12012-019-09516-x (2019). 30963443 10.1007/s12012-019-09516-x PMC6746878

[68] Ben Taleb, Z. et al. Pod-based e-cigarettes versus combustible cigarettes: the impact on peripheral and cerebral vascular function and subjective experiences. *Tob. Induc. Dis.* **21**, 71. 10.18332/tid/162366 (2023). 37252033 10.18332/tid/162366 PMC10210091

[69] Ashraf, M. T. et al. Association between e-cigarette use and myocardial infarction: a systematic review and meta-analysis. *Egypt. Heart J.* **75**, 97. 10.1186/s43044-023-00426-6 (2023). 38032522 10.1186/s43044-023-00426-6 PMC10689622

[70] Lee, W. H. et al. Modeling cardiovascular risks of E-Cigarettes with Human-Induced pluripotent stem Cell-Derived endothelial cells. *J. Am. Coll. Cardiol.* **73**, 2722–2737. 10.1016/j.jacc.2019.03.476 (2019). 31146818 10.1016/j.jacc.2019.03.476 PMC6637948

[71] Brown, A. E., Carpenter, M. J. & Sutfin, E. L. Occasional smoking in college: who, what, when and why? *Addict. Behav.* **36**, 1199–1204. 10.1016/j.addbeh.2011.07.024 (2011). 21849231 10.1016/j.addbeh.2011.07.024 PMC3179822

[72] GBD 2019 Tobacco Collaborators. Spatial, temporal, and demographic patterns in prevalence of smoking tobacco use and attributable disease burden in 204 countries and territories, 1990-2019: a systematic analysis from the Global Burden of Disease Study 2019. Lancet 397, 2337-2360. https://doi.org:10.1016/s0140-6736(21)01169-7 (2021). 10.1016/S0140-6736(21)01169-7 PMC8223261 34051883

