# Supplementary material for: Disposable e-cigarettes and their nicotine delivery, usage pattern, and subjective effects in occasionally smoking adults
Source: Sci Rep. 2025 May 9;15:16270. doi: 10.1038/s41598-025-97491-5 (PMC12064830; doi:10.1038/s41598-025-97491-5)
Supplement: Supplementary file 1 — Supplementary Material 1 [file 41598_2025_97491_MOESM1_ESM.pdf]

SUPPLEMENT

Detailed study hypotheses:

- The Elf Bar 600 makes it easier for non-smokers and occasional smokers to start smoking (compared to myblu and tobacco cigarettes)
- Smoke inhalation differs between products in terms of volume and draw duration
  - The average draw volume is greater for Elf Bar Strawberry-Kiwi than Elf Bar Tobacco, followed by myblu and tobacco cigarette
  - Average puff duration is highest for Elf Bar Strawberry-Kiwi, followed by Elf Bar Tobacco, myblu, and tobacco cigarette
- The puff frequency (puffs/time) is highest for Elf Bar Strawberry-Kiwi, followed by Elf Bar Tobacco, myblu, and tobacco cigarette
- The number of average puffs per measurement duration is highest for Elf Bar Strawberry-Kiwi, followed by Elf Bar Tobacco, myblu and tobacco cigarette
- The nicotine flooding is equally fast for the tobacco cigarette and the Elf bar Strawberry-Kiwi when used ad libitum, followed by Elf bar tobacco and then the myblu
- When using the tobacco cigarette and the Elf Bar Strawberry-Kiwi, the highest maximum nicotine concentrations are reached, followed by Elf Bar tobacco and then by myblu
- The smoking experience is perceived more positively with Elf Bar Strawberry-Kiwi than with Elf Bar Tabak, followed by myblu and tobacco cigarette
- The smoking taste is perceived more positively with Elf Bar Strawberry-Kiwi than with Elf Bar Tabak, followed by myblu and tobacco cigarette
- The motivation to consume another product after finishing the consumption is greater with Elf Bar Strawberry-Kiwi than with Elf Bar Tobacco, followed by myblu and tobacco cigarette
- Cardiovascular effects (on blood pressure, heart rate, vascular stiffness) increase in a dose-dependent manner and are more pronounced with tobacco cigarettes and Elf Bar Strawberry-Kiwi than with Elf Bar Tobacco, followed by myblu
- The side effect profile is least pronounced in number, strength and duration to the qualities mentioned below with myblu, followed by Elf Bar Tobacco and Elf Bar Strawberry-Kiwi, with tobacco cigarettes the side effect profile is most pronounced

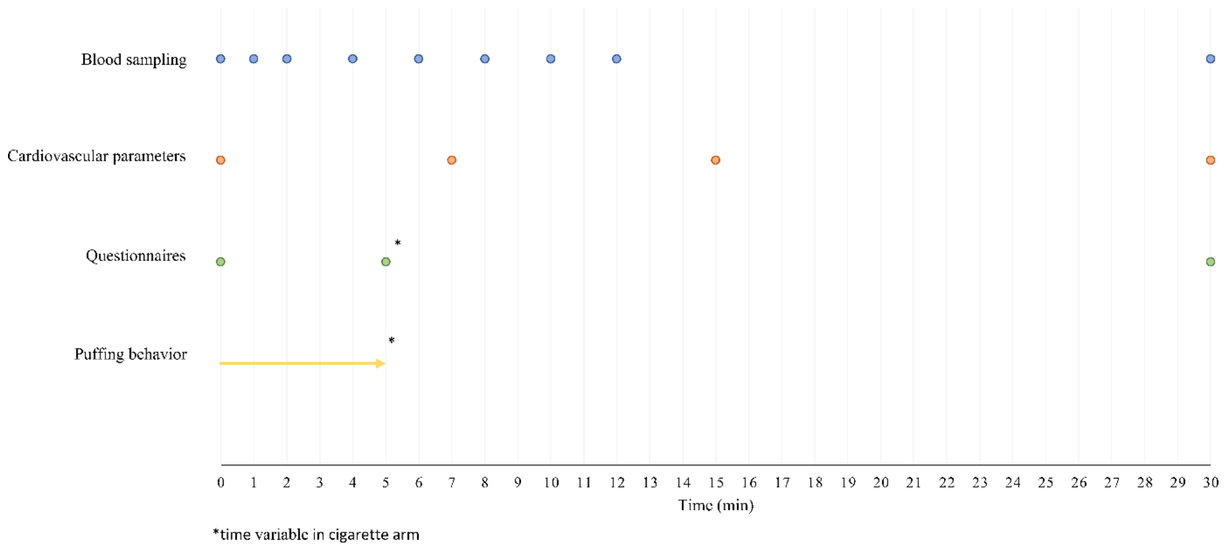

Figure S1. Study design overview with time points and their measurements

Additional information on methods:

The criteria of inclusion:

- between 18 and 29 years old
- lifetime-smoker (≥ 100 cigarettes smoked)
- self-declaration as occasional smoker (smoking at least once a month but not more than 3 days per week)
- nicotine abstinence at least 12 hours prior to an examination
- nicotine concentration of venous plasma under 10 ng/ml immediately before an examination
- capacity to consent

The criteria of exclusion:

- under 18 or over 29 years old
- non-smoker

- tobacco dependence according to ICD-10/FTND (Fagerström Test for Nicotine Dependence) > 1 or PSECD (Penn State Electronic Cigarette Dependence Index) > 3
    - daily cigarette smoking
    - frequent consumption of other nicotine products
- Current pregnancy
    - for contraceptive methods with Pearl index > 0.9, a negative pregnancy test must be available before starting testing
- pre-existing psychiatric conditions
    - acute suicidality
    - acute psychiatric illness as defined by ICD-10
    - drug-, medication- or alcohol abuse at the time of the study
- pre-existing internal diseases
    - acute malignant cancer
    - malignant cancer in the past 5 years
    - severe pre-existing conditions/risk factors of the cardiovascular system
        - manifest arterial hypertension
        - severe cardiac history (history of myocardial infarction, heart failure)
        - cardiac pacemaker
        - diabetes mellitus
    - respiratory disease (such as asthma, COPD)
    - severe active infectious diseases (hepatitis, HIV)
    - CO > 5ppm in the expiratory fluid
    - Other circumstances that, in the opinion of the investigator, militate against participation in the study

Table S1. Personal characteristics of participants included in this study.

Age, mean (standard deviation (SD) and range)	24 ± 2.1 (19-28)
Sex, female, n (%)	9 (50 %)
Sex, male, n (%)	9 (50 %)
Height in cm, mean (SD)	176.5 ± 7.8
Weight in kg, mean (SD)	71.6 ± 12
Number of days on which subjects smoked within the last 30 days, mean (SD, range)	4.1 ± 2.6 (1-10)
Number of days on which subjects smoked within the last 6 months, mean (SD, range)	28.7 ± 12.1 (10-50)

A total number of 32 individuals were screened, 14 of them were excluded: three met an exclusion criterion, two dropped out by mutual agreement, nine had to be excluded due to technical problems (seven with blood sampling as PK analysis was not possible, two due to early stop of recording with the SPA-M device).

Among included participants, seven indicated occasional consumption of nicotine products in addition to occasionally smoking cigarettes: three used disposable ECs, two cannabis products, one a hookah, and one snus. One additional person had tried disposable ECs before once or twice. None of those occasionally using ECs were considered dependent according to PSECDI.

To secure a tobacco abstinence of at least 12 hours, the participants' CO in the exhaled air was measured. Afterward, the calibration measurement of cardiovascular parameters was performed. When necessary (woman with the use of a contraceptive method with a Pearl Index > 0.9), a pregnancy test was taken. Then the participant filled out two questionnaires (Modified Cigarette Evaluation Questionnaire (mCEQ); Effect comparison after consumption (ENK)) and venous access was established. The baseline blood sample was drawn and the baseline cardiovascular parameters were measured. Afterward, the participant received the product.

The mCEQ examines subjective effects of smoking. The English version is validated for its dimensions [2]. We used a German version that was previously translated by our study group.

Minor rewordings were needed to adapt the questionnaire for nicotine products other than cigarettes. Adapted versions of the mCEQ have been successfully used in other studies (e.g., [3]). The questionnaire contains 12 items divided into three multi-item subscales (measuring smoking satisfaction, psychological reward, and aversion) and two single items (measuring enjoyment of respiratory tract sensations and craving reduction). Each item can be rated on a 7-point Likert-type scale (1 = does not apply at all, 2 = does not apply, 3 = does rather not apply, 4 = neutral, 5 = does rather apply, 6 = does apply, 7 = completely right). Higher scores imply a higher expression on the respective scale.

The ENK served to assess the subjective side effects of smoking/EC use. On a scale from 1 to 10, participants rated drowsiness, mouth irritation, throat irritation, dizziness, salivation, cold hands/feet, heart pounding, headache, sweating, nausea, feeling of vomiting, nicotine flash, and other effects if applicable. The ENK was used with slightly varying items in previous studies published by our study group [4-6].

Each questionnaire was filled out before consumption, after consumption, and 30 min after starting the consumption. Moreover, right after smoking or EC use, the participants had to rate their motivation to directly consume the tested product again on a scale from 1 to 7 (1 = does not apply at all, 4 = neutral, 7 = completely right).

Blood samples were kept between 4 to 8 °C until they were centrifuged and an internal standard solution was added to the plasma. Storage until shipment on dry-ice was at - 80 °C. The further analysis took place in Berlin, Germany at the German Federal Institute for Risk Assessment (BfR) with a validated analytical method using liquid chromatography with tandem mass spectrometry (LC-MS/MS) after protein precipitation [7].

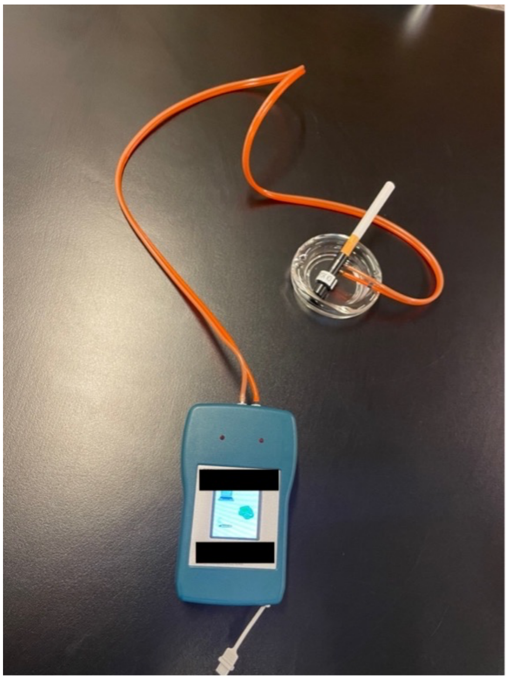

Figure S2. Smoking Puff Analyzer – Mobile (SPA-M) device

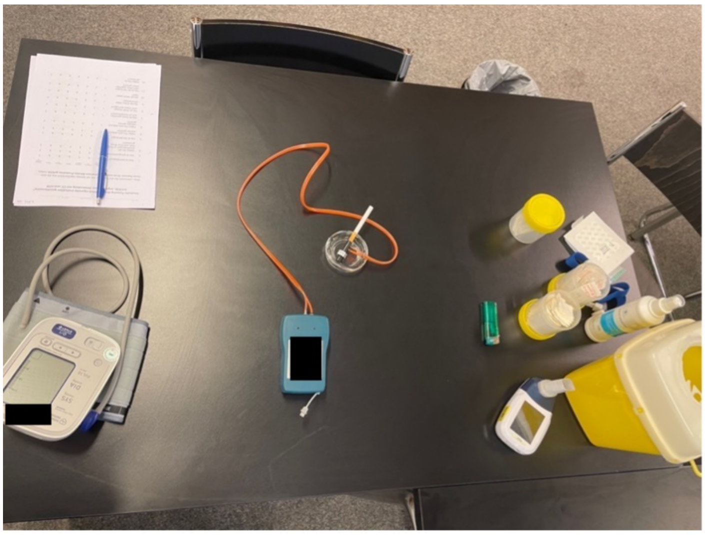

Figure S3. Study setup

Table S2. Summary of p-values for puffing behavior.

Compared products	Disposable EC strawberry-kiwi vs. Disposable EC tobacco	Disposable EC strawberry-kiwi vs. Cigarette	Disposable EC strawberry-kiwi vs. Pod EC	Disposable EC tobacco vs. Cigarette	Disposable EC tobacco vs. Pod EC	Cigarette vs. Pod EC
Average puff volume	0.59	<0.05	0.46	<0.05	0.72	<0.05

Average puff duration	<0.05	<0.05	0.99	<0.05	0.23	<0.05
Average flow per puff	0.72	<0.05	0.93	<0.05	0.59	<0.05
Average peak flow per puff	0.83	<0.05	0.7	<0.05	0.42	<0.05
Puff frequency	0.41	<0.05	0.59	<0.05	0.62	<0.05
Average interval between puffs	0.87	<0.05	0.76	<0.05	0.88	<0.05
Total duration	0.95	<0.05	0.54	<0.05	0.41	<0.05
Total number of puffs	0.42	0.17	0.78	0.07	0.43	0.18

Table S3. Summary of p-values for mCEQ subscale results.

Compared products	Disposable EC strawberry-kiwi vs. Disposable EC tobacco	Disposable EC strawberry-kiwi vs. Cigarette	Disposable EC strawberry-kiwi vs. Pod EC	Disposable EC tobacco vs. Cigarette	Disposable EC tobacco vs. Pod EC	Cigarette vs. Pod EC
Satisfaction	<0.05	<0.05	<0.05	<0.05	<0.05	0.63
Enjoyment of respiratory tract sensations	0.7	<0.05	<0.05	0.42	0.06	0.1
Psychological reward	0.26	0.3	0.36	0.97	0.89	0.97
Craving reduction	0.39	0.54	0.43	0.13	0.16	0.79
Aversion	0.77	0.28	0.51	0.09	0.53	<0.05

Table S4. Motivation to directly consume the tested product again (measured directly after the end of consumption) in mean with 95% CI.

Product	Disposable EC strawberry-kiwi	Disposable EC tobacco	Cigarette	Pod EC
Score	2.9 (2.1-3.7)	2.7 (1.9-3.5)	1.3 (1-1.7)	1.5 (1-2)

Table S5. Summary of p-values for motivation to directly consume the tested product again.

Compared products	Disposable EC strawberry-kiwi vs. Disposable EC tobacco	Disposable EC strawberry-kiwi vs. Cigarette	Disposable EC strawberry-kiwi vs. Pod EC	Disposable EC tobacco vs. Cigarette	Disposable EC tobacco vs. Pod EC	Cigarette vs. Pod EC
P-value	0.7	<0.05	<0.05	<0.05	<0.05	0.56

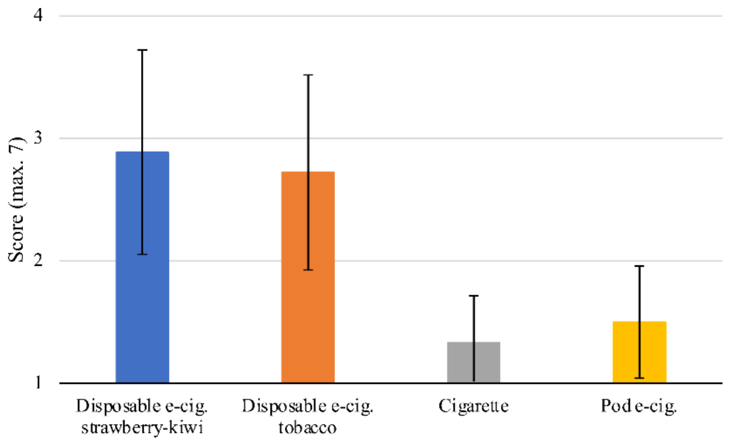

Figure S4. Motivation to directly use the tested product again (mean and 95% CI)

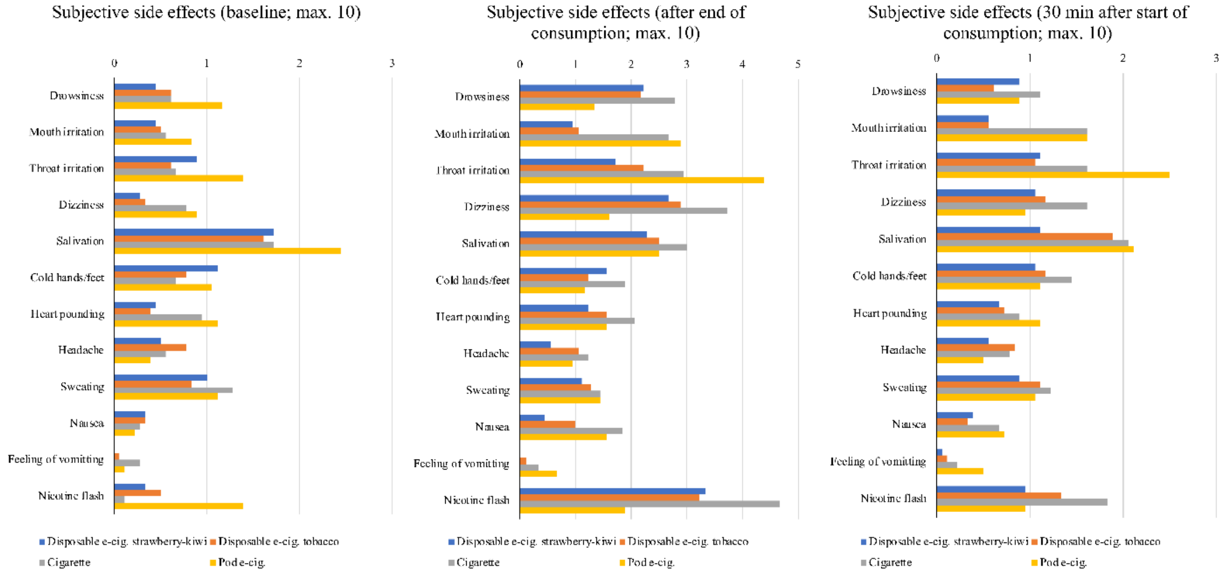

Figure S5. Summary of results regarding subjective side effects (mean).

Additional information on side effects:

Four minor adverse events (circulatory problems) occurred: Three related to placing of the venous access (with one of them reoccurring after the end of disposable EC tobacco consumption) and one about 3 min after the end of cigarette smoking during blood sampling. Two of which led to exclusion because of too many missing blood samples.

1. Bondy SJ, Victor JC, Diemert LM. Origin and use of the 100 cigarette criterion in tobacco surveys. *Tob Control* 2009;18(4):317-23. doi: 10.1136/tc.2008.027276 [published Online First: 20090601]
2. Cappelleri JC, Bushmakin AG, Baker CL, et al. Confirmatory factor analyses and reliability of the modified cigarette evaluation questionnaire. *Addictive Behaviors* 2007;32(5):912-23. doi: https://doi.org/10.1016/j.addbeh.2006.06.028
3. Hatsukami DK, Zhang Y, O'Connor RJ, et al. Subjective responses to oral tobacco products: scale validation. *Nicotine Tob Res* 2013;15(7):1259-64. doi: 10.1093/ntr/nts265 [published Online First: 20121213]
4. Vukas J, Mallock-Ohnesorg N, Rüther T, et al. Two Different Heated Tobacco Products vs. Cigarettes: Comparison of Nicotine Delivery and Subjective Effects in Experienced Users. *Toxics* 2023;11(6) doi: 10.3390/toxics11060525 [published Online First: 20230611]
5. Rabenstein A, Rahofer A, Vukas J, et al. Usage Pattern and Nicotine Delivery during Ad Libitum Consumption of Pod E-Cigarettes and Heated Tobacco Products. *Toxics* 2023;11(5) doi: 10.3390/toxics11050434 [published Online First: 20230505]
6. Mallock N, Rabenstein A, Gernun S, et al. Nicotine delivery and relief of craving after consumption of European JUUL e-cigarettes prior and after pod modification. *Sci Rep* 2021;11(1):12078. doi: 10.1038/s41598-021-91593-6 [published Online First: 20210608]
7. Mallock N, Rabenstein A, Laux P, et al. Rapid, sensitive, and reliable quantitation of nicotine and its main metabolites cotinine and trans-3'-hydroxycotinine by LC-MS/MS: Method development and validation for human plasma. *J Chromatogr B Analyt Technol Biomed Life Sci* 2021;1179:122736. doi: 10.1016/j.jchromb.2021.122736 [published Online First: 20210505]
